# A Study of Antibiotic Tolerance to Levofloxacin and Rifampin in *Staphylococcus aureus* Isolates Causing Prosthetic Joint Infections: Clinical Relevance and Treatment Challenges

**DOI:** 10.3390/antibiotics15010010

**Published:** 2025-12-20

**Authors:** María Ángeles Meléndez-Carmona, Irene Muñoz-Gallego, Mikel Mancheño-Losa, Jaime Lora-Tamayo

**Affiliations:** 1 Department of Clinical Microbiology, Instituto de Investigación Biomédica “i+12”, Hospital Universitario 12 de Octubre, 28041 Madrid, Spain; mariadelosangeles.melendez@salud.madrid.org (M.Á.M.-C.); irene.munoz@salud.madrid.org (I.M.-G.); 2 Spanish Study Group on Bone and Joint Infections (GEIO-SEIMC), 28003 Madrid, Spain; mikel.mancheno@salud.madrid.org; 3 Department of Internal Medicine, Instituto de Investigación Biomédica “i+12”, Hospital Universitario 12 de Octubre, 28041 Madrid, Spain; 4 CIBER Enfermedades Infecciosas, Instituto de Salud Carlos III, 28029 Madrid, Spain

**Keywords:** prosthetic joint infection, implant-associated infection, antimicrobial resistance, biofilm

## Abstract

**Background**: Antibiotic tolerance in *Staphylococcus aureus* biofilms poses a major clinical challenge in prosthetic joint infections (PJIs). This study aimed to characterize the antibiotic tolerance of clinical *S. aureus* isolates recovered from cases of PJI under different stress conditions, including biofilm formation and antibiotic exposure. The correlation between tolerance level, the presence of specific tolerance-related genes, and clinical outcome was also evaluated. **Methods**: Twelve clinical *S. aureus* isolates were analyzed. To assess tolerance, the TDtest was used on exponentially growing bacteria, 48 h biofilms, and biofilms treated with levofloxacin and/or rifampin. Whole-genome sequencing was performed to identify tolerance-associated genes. **Results**: All isolates were phenotypically susceptible to rifampin and levofloxacin. Although all strains exhibited basal tolerance levels, biofilm formation led to heightened antibiotic tolerance, particularly those treated with rifampin as compared to levofloxacin: 29.5 vs. 17 (*p* = 0.01). Rifampin tolerance in biofilm-embedded bacteria was significantly higher in isolates from patients with treatment failure (*p* < 0.0001). Levofloxacin tolerance showed no significant association with clinical outcomes. There was no correlation between reduction in biofilm bacterial burden after treatment and tolerance levels. Genomic analysis identified associations between higher levofloxacin tolerance and the presence of *sspA* and *leuS* in biofilm isolates, and between rifampin tolerance and *prs* and *pgm*. **Conclusions**: In this study, clinical *S. aureus* strains isolated from prosthetic joint infections exhibited considerable inter-strain variability in antibiotic tolerance, particularly under biofilm conditions. Elevated rifampin tolerance in biofilm-embedded bacteria was associated with poor clinical outcomes, underscoring the need for tolerance assessment beyond standard susceptibility testing.

## 1. Introduction

Antibiotic tolerance in biofilms is a critical factor in the treatment of implant-associated infections. Tolerance is defined as the ability of a bacterial population to survive transient exposure to high antibiotic concentrations without altering its minimum inhibitory concentration (MIC) [1]. It must be differentiated from persistence, where the ability to survive during a period of time applies to specific bacterial subpopulations, and to resistance, where a MIC increase is observed and the bacterial population may survive indefinitely despite antibiotic exposure [1,2]. The phenomena of tolerance is to be expected in biofilm-embedded bacteria, and so in any foreign-body associated infection, which makes these bacterial infections more difficult to treat and may also contribute to their chronicity [3]. Regardless of the results of a standard antibiogram, a tolerant population is assumed to be present on clinical grounds. Consequently, tolerant strains may be incorrectly classified as fully susceptible, resulting in treatment failure [4,5].

In this regard, prosthetic joint infections (PJIs) are considered difficult to treat, primarily because the bacteria that cause them often form biofilms on the device surface [6]. *Staphylococcus aureus* is the single most frequent etiology of acute PJI (i.e., early post-surgical and hematogenous infections) [7], where a management with debridement, antibiotics, and implant retention may be considered [6,8]. In this clinical context, the current recommended therapy is a combination of rifampin (RIF) plus a fluoroquinolone (such as levofloxacin [LVX]) [6,8,9]. Rifampin-based combinations have indeed shown in pre-clinical models (in vitro and in vivo) and in clinical research to overcome many of the handicaps posed by foreign-body infections where antimicrobial tolerance is expressed [10].

However, antimicrobial inefficacy of levofloxacin and rifampin may still be observed. Antimicrobial tolerance varies depending on several factors, including the given antimicrobial agents used, the specific bacterial strain and species, and the growth conditions and maturity (i.e., time of growth) of the biofilm [2,11].

The specific bacterial strain genetic background may indeed influence in the tolerance expression in a given infection. Genetic determinants play a crucial role in bacterial persistence and antibiotic tolerance. Several genes previously described in the literature have been associated with tolerance mechanisms, potentially influencing treatment outcomes [12]. However, their clinical relevance is still being studied.

Our research group recently investigated the phenotypic and genotypic characteristics that contribute to the persistence or recurrence of PJIs caused by *Staphylococcus aureus* [13]. In a parallel study, we also explored variability in response to levofloxacin and rifampin, using two different experimental models [14]. The aim of the present study was to build upon these findings by assessing the tolerance of a clinical collection of *S. aureus* isolates under different stress conditions, including biofilm formation and antibiotic exposure. We have also evaluated whether the presence or absence of certain representative tolerance-related genes correlated with tolerance levels as measured by the TDtest. This integrative approach seeks to provide a deeper understanding of the genetic basis of antibiotic tolerance in *S. aureus* causing PJI and its potential clinical implications. While the clinical correlation of in vitro models is not straightforward [15] the TDtest is a simple model that has allowed us to compare the tolerance of several clinical strains of *S. aureus* producing PJI

## 2. Results

### 2.1. Antimicrobial Susceptibility

Table 1 and Table 2 summarize the TDtest experiments on tolerance to levofloxacin and rifampin among the 12 *S. aureus* isolates analyzed in this study. According to the MIC data, all isolates were susceptible to both antimicrobials.

### 2.2. Tolerance of S. aureus Isolates to Levofloxacin and Rifampin Under Different Stress Conditions

**Figure 1:**
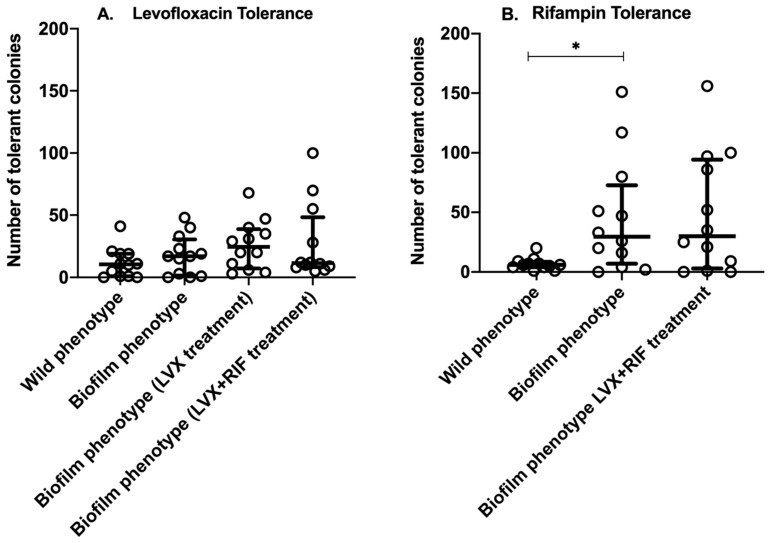
Number of tolerant colonies of 12 *S. aureus* isolates included under different stress conditions. Measured tolerance by TDtest method to levofloxacin (LVX) (**A**) and to rifampin (RIF) (**B**). Tolerance was expressed as the number of colonies present within the inhibition zone. Measurement of rifampin-tolerance was not possible after treatment with rifampin-monotherapy, since all strains developed resistance within 24 h. Each dot represents the mean of three replicates of each isolate. Bar denotes median with interquartile range of aggregate data. * *p* < 0.05.

On exponentially growing (non-biofilm) *S. aureus* isolates, the tolerance disk test (TDtest) revealed a median of 10.5 [IQR: 1–19] levofloxacin-tolerant colonies and 6 [IQR: 4–8.5] rifampin-tolerant colonies per inhibition zone (Figure 1). No statistically significant differences were found between the two antibiotics (*p* = 0.4) and none of the 12 isolates showed high-level tolerance (>100 colonies).

After 48 h of biofilm growth, the median biofilm density reached 5.7 log_10_ CFU/cm^2^ [IQR: 5.5–5.8]. The number of tolerant colonies under 48 h biofilm conditions was higher for both antibiotics when compared to non-biofilm isolates. Although the difference was not statistically significant (*p* = 0.06), levofloxacin tolerance increased a median of 17 colonies [IQR: 1.5–30.5]. Rifampin tolerance increased markedly under these conditions, reaching a median of 29.5 colonies [IQR: 7–72.8]. This difference was statistically significant (*p* < 0.0001). A comparison of the two antibiotics under 48 h biofilm conditions revealed that rifampin induced a significantly higher number of tolerant colonies than levofloxacin (29.5 [IQR: 7–72.8] vs. 17 [IQR: 1.5–30.5], *p* = 0.01).

After 24 h of antimicrobial treatment with levofloxacin and/or rifampin 48 h-biofilms, the residual bacterial density was 2.3 log_10_ CFU/cm^2^ [IQR: 1.8–2.6] for levofloxacin, 4.7 log_10_ CFU/cm^2^ for rifampin [IQR: 4.4–4.9], and 3.1 log_10_ CFU/cm^2^ [IQR: 2.5–3.6] for the combination. Regarding levofloxacin tolerance, no significant differences were found between untreated and combination-treated biofilms (median 17 [IQR: 1.5–30.5] vs. 11.5 [IQR: 8.3–48.3], *p* = 0.8). The number of tolerant colonies observed with levofloxacin monotherapy was higher than with the combination (24.5 [IQR: 7.3–38.8] vs. 11.5 [IQR: 8.3–48.3]), but the difference was not statistically significant (*p* = 0.3).

After 24 h of rifampin monotherapy, all biofilms showed complete resistance in the disk diffusion assay, with inhibition zones shrinking to 0 mm. There were no significant differences in the number of tolerant colonies between untreated biofilms (29.5 [IQR: 7–72.6]) and biofilms treated with levofloxacin plus rifampin (30 [IQR: 3–94.3], *p* = 0.9) Furthermore, no significant differences in tolerance to rifampin and levofloxacin were observed with the rifampin-levofloxacin combination (30 [IQR: 3–94.3] vs. 11.5 [IQR: 8.3–48.3], *p* = 0.1). Supplementary Figure S2 shows the development of tolerant colonies for each specific strain.

Of note, in all types of stress used herein to detect tolerance, a high dispersion of results was observed among the strains analyzed, with a very high inter-strain coefficient of variability (Table 1).

**Figure 2:**
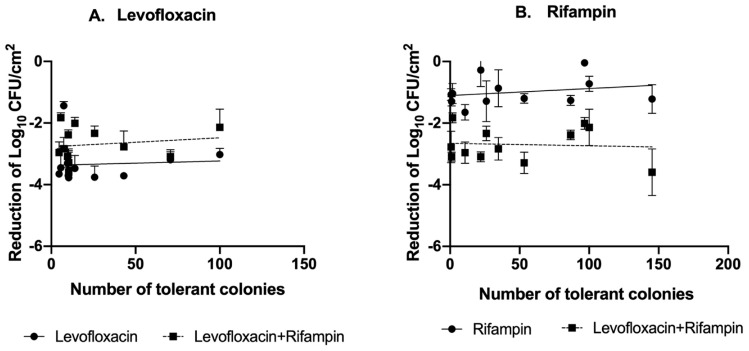
Correlation between bacterial tolerance (TDtest) and log10 CFU/cm^2^ variation under different antibiotic treatments. The figure illustrates the correlation between the number of tolerant colonies to (**A**) levofloxacin and (**B**) rifampin and the reduction in bacterial load (log_10_ CFU) following treatment with levofloxacin, rifampin and their combination using the static in vitro biofilm model. The reduction is calculated as the log_10_ CFU/cm^2^ after 24 h of antimicrobial treatment (end of treatment)—the log_10_ CFU/cm^2^ after 48 h of biofilm growing (beginning of treatment). (**A**)**.** Spearman’s rho = 0.06 (95% CI: −0.5 to 0.6, *p* = 0.8) under LVX monotherapy and 0.2 (95% CI: −0.5 to 0.7, *p* = 0.6) under combination therapy. (**B**)**.** Spearman’s rho = 0.2 (95% CI: −0.4 to 0.7, *p* = 0.5) under RIF monotherapy and −0.1 (95% CI: −0.6 to 0.5, *p* = 0.8) under combination therapy. Bar denotes mean with standard deviation of each isolate.

Finally, correlation analysis revealed no significant association between the number of tolerant colonies and the reduction in biofilm-embedded bacteria after treatment with either drug (Figure 2).

**Figure 3:**
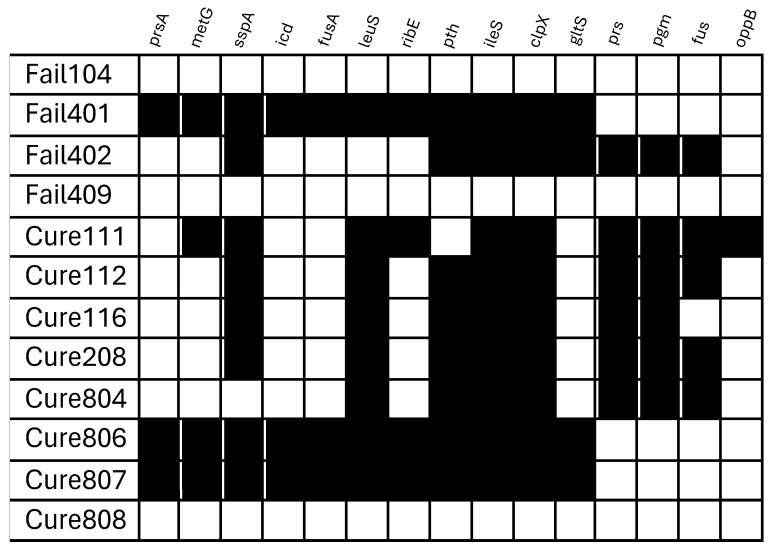
Presence and absence of tolerance-associated genes in *S. aureus* isolates. This figure illustrates the distribution of analyzed genes among the 12 *S. aureus* isolates from prosthetic joint infections. Each row represents a bacterial strain, while each column corresponds to a specific gene. Black squares indicate the presence of the gene, whereas white squares denote its absence.

Figure 3 shows the distribution of selected genes across the *S. aureus* isolates analyzed. Among the genes studied, significant associations were under two specific conditions. First, in 48 h biofilm isolates, the presence of *sspA* (number of tolerant CFU: 41.3 ± 8.7 vs. 18.4 ± 5.5, *p* = 0.04) and *leuS* (44.6 ± 8.2 vs. 16.7 ± 4.4, *p* = 0.008) was significantly correlated with higher levofloxacin tolerance levels as measured by the TDtest. Second, in exponentially growing (non-biofilm) isolates, tolerance to rifampin was significantly associated with the presence of *prs* and *pgm*; for both genes, number of tolerant CFU was as follows: 12.3 ± 3.5 vs. 3.5 ± 0.7, *p* = 0.03.

### 2.3. Tolerance According to Clinical Prognosis

**Figure 4:**
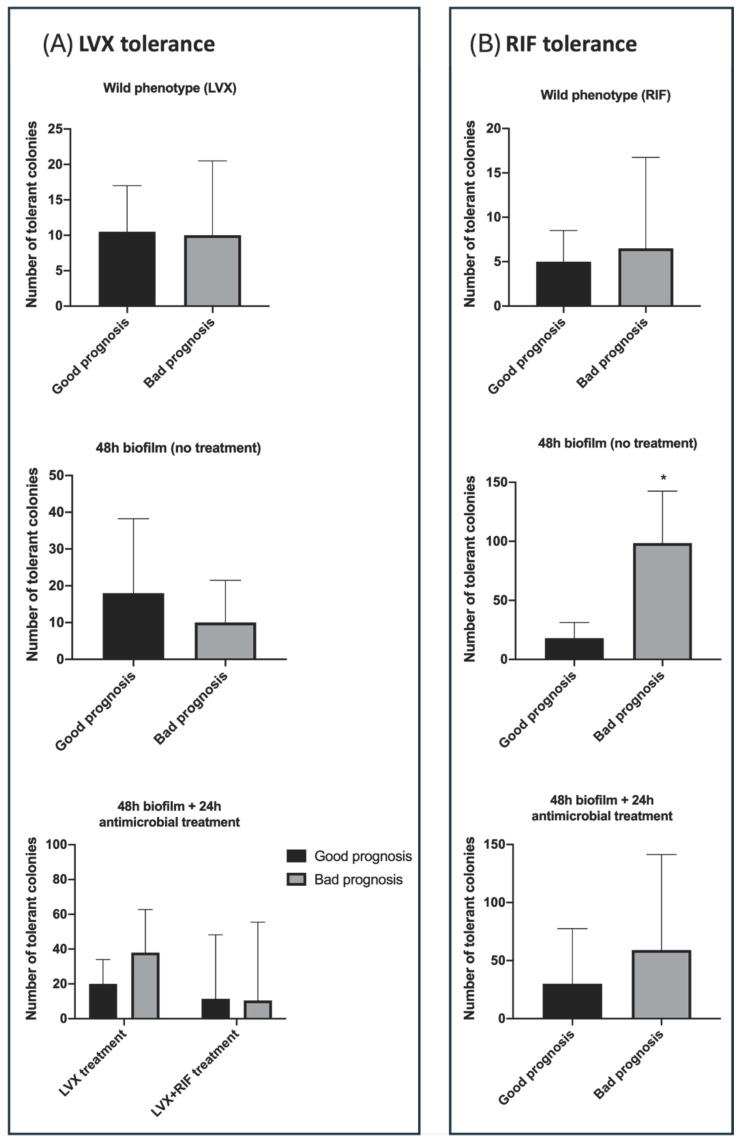
Aggregated data on tolerance levels to levofloxacin (LVX) and rifampin (RIF) according to prognosis measured by TDtest method in the 12 *S. aureus* isolates included in the study. (**A**) Number of tolerant bacterial colonies in different stress conditions to levofloxacin (LVX), (**B**) Number of tolerant bacterial colonies in different stress conditions to rifampin (RIF). All values are expressed as median of three replicates, the bar denotes the interquartile range (IQR). * *p* < 0.05.

Under the exponentially growing (non-biofilm) conditions, no significant differences in tolerance to levofloxacin or rifampin were observed when comparing isolates from patients with favorable outcomes to those with treatment failure. The median number of levofloxacin-tolerant colonies was 10.5 [IQR: 2–17] versus 10 [IQR: 0.3–20.5] (*p* = 0.9); the median number of rifampin-tolerant colonies was 5 [IQR: 1.8–8.5] log_10_ CFU versus 6.5 [IQR: 5.3–16.8] log_10_ CFU (*p* = 0.08). In biofilm-embedded bacteria, rifampin tolerance was significantly higher in isolates from patients with prosthetic joint infection (PJI) failure compared to those with favorable outcomes (median 98.5 [IQR: 55.3–142.5] vs. 18 [IQR: 2.5–31.3], *p* < 0.0001). Meanwhile levofloxacin tolerance showed no significant differences (median 10 [IQR: 0.8–21.5] vs. 18 [IQR: 4.5–38.3], *p* = 0.1). After combination therapy, tolerance levels for both antibiotics remained comparable between outcome groups. For levofloxacin, the number of tolerant colonies was 10.5 [IQR: 6.8–55.5] vs. 11.5 [IQR: 8.5–48.3] (*p* = 0.9) and for rifampin, 59 [IQR: 12–141.3] vs. 30 [IQR: 0.3–77.5] (*p* = 0.09) (Figure 4). Finally, the presence or absence of specific genetic markers in the isolates was not significantly associated with clinical prognosis.

## 3. Discussion

In this study, we evaluated tolerance to levofloxacin and rifampin in 12 *S. aureus* strains isolated from patients with PJI. Antibiotic tolerance in *S. aureus* is a serious concern in clinical microbiology, especially in the context of biofilm-associated infections [2]. For our study, we applied the tolerance disk test, which has been used by various authors to assess persistence and tolerance [16,17], directly to *S. aureus* colonies.

All strains exhibited basal tolerance, but the level varied significantly between strains, showing a very high coefficient of inter-strain variability, suggesting again the importance of each specific strain when addressing a given infection.

There was a significant increase in the number of tolerant colony-forming units when bacteria were recovered from 48 h biofilms, especially after being treated with rifampin. Variance according to the underlying stress is consistent with the phenotypic nature of tolerance, which typically develops during biofilm formation. This finding is also consistent with previous studies that have emphasized the protective role of the biofilm matrix in reducing antimicrobial efficacy [18,19]. Of note, the biofilm formation was the most significant source of stress. It led to the greatest increase in the number of tolerant CFUs, surpassing the effect observed after exposure to antibiotics. However, it is also important to acknowledge that the antimicrobial concentrations were above the MIC and that we used a static in vitro model (meaning that the concentrations would not decrease over time). Our study did not address whether tolerance would increase under sub-MIC antibiotic concentrations. Overall, these observations highlight the phenomenon of tolerance in a representative clinical collection of staphylococcal isolates and emphasize the importance of specific strain properties when considering clinical presentation and outcome in implant-associated staphylococcal infections such as PJI [14].

We also observed that rifampin tolerance was greatest among biofilm-embedded strains recovered from PJI patients who had poor clinical outcomes. This was not observed among non-biofilm bacteria, which underscores both the importance of measuring bacterial antimicrobial tolerance under specific stress conditions, as well as the potential for integrating tolerance assays into routine susceptibility testing, particularly for biofilm-associated infections. The latter finding is interesting, since rifampin remains the backbone therapy for staphylococcal PJIs, especially when managed with implant retention [10]. A high number of rifampin-tolerant, biofilm-embedded bacteria could be interpreted as a clinical warning sign of a PJI case at risk of failure. Whether these patients would require an alternative to a rifampin-based combination therapy or would fail anyway is a matter for further studies [20]. In fact, this differential increase in the number of rifampin-tolerant bacteria was conspicuously tempered when biofilms were treated with rifampin plus levofloxacin, which reinforces the usefulness of the combination therapy. Unlike rifampin, however, levofloxacin tolerance was not associated with clinical failure, which could reflect the good antibiofilm activity of the later fluoroquinolone generations (mainly levofloxacin and moxifloxacin), as noted by other authors [21].

Regardless of the overall clinical prognosis, we found no association between the antibiofilm efficacy of the antimicrobial regimens used (measured by the decrease in biofilm bacterial density after 24 h of exposure to antibiotics) and the number of tolerant strains tolerant to either rifampin or levofloxacin. The TDtest is an in vitro technique used to identify and ‘resuscitate’ tolerant bacteria that survive exposure to bactericidal concentrations of antibiotics [16]. However, as has been observed with other microbiological indexes when applied to biofilm-embedded bacteria, such as the MIC or the minimal biofilm eradication concentration (MBEC) [14,22], the TDtest is unable to predict the antibiofilm efficacy of a given antimicrobial treatment.

Our analysis of tolerance-associated genes revealed new insights into the genetic factors influencing bacterial persistence under antimicrobial stress. While most of the analyzed genes were not significantly correlated with tolerance levels measured by the TDtest, we identified specific associations under certain conditions. Notably, the presence of *sspA* and *leuS* genes in isolates recovered from 48 h biofilms was significantly correlated with higher levofloxacin tolerance, suggesting a possible role in biofilm-associated persistence mechanisms [23]. Among non-biofilm isolates, rifampin tolerance was significantly associated with the presence of *prs* and *pgm*, indicating the possible contribution of these genes to intrinsic tolerance mechanisms [24].

Our study has some limitations that need to be addressed. First, while the TDtest provides valuable data on antibiotic tolerance, its methodological constraints may not fully capture the complex dynamics of bacterial tolerance in vivo. Indeed, the specific clinical conditions under which the infection develops, such as chronicity, biofilm maturity, host–pathogen interactions, the intracellular environment, and the consequences of surgery, as well as the heterogeneity and complexity of biofilm architecture, cannot be reproduced by this in vitro model. This limitation could affect the generalizability of our results to real-world PJI cases, where biofilm growth conditions are highly variable. Second, the study was limited to 12 clinical isolates of *S. aureus* obtained from acute PJIs. While these isolates represent a relevant spectrum of clinical presentations and outcomes of staphylococcal PJIs, the relatively small number of strains studied limits the statistical power of our study. Also, some imbalance of cases with good and bad prognosis came out of the random selection, but this avoided a selection bias. Because we did not apply statistical corrections for multiple comparisons, particularly when analyzing the genetic background of the strains, we cannot rule out that some significant observations occurred by chance. Third, although tolerance is a dynamic phenotypic process, the study did not investigate the underlying transcriptomic and molecular mechanisms of our findings. Fourth, in our study, we utilized a fixed concentration of antibiotics with static exposure over 24 h, which may not accurately represent the pharmacokinetics and pharmacodynamics of antibiotic therapy in vivo, where drug concentrations fluctuate over time and bacterial exposure to subinhibitory concentrations may also influence the development of tolerance. Likewise, our analysis of the increase in tolerance among biofilm-embedded bacteria focused on 48 h-old mature biofilms and did not consider tolerance in chronic or more mature biofilms and their interaction with antimicrobial treatment. Fifth, while a higher rate of rifampin tolerance was observed with some stresses, it was not possible its study with the use of rifampin alone due to the rapid development of resistance. Sixth, we did not specifically assess that MIC did not increase among tolerant CFU recovered after the second phase of the TDtest. However, the TDtest is a validate method for detecting tolerance, where by definition no increase in MIC is observed [1,16]. Finally, the evaluation of tolerance in bacteria recovered from biofilms needed an overnight culture before starting the TDtest that could account for a loss of biofilm-associated properties, including tolerance. However, it is important to remark that the full recovery of planktonic properties after a phase of biofilm and sessile state is not immediate, but may take some time [2], and we did observe differences regarding the number of tolerant bacteria between the wild (i.e., exponentially growing) and the biofilm phenotype.

In conclusion, this study explores the challenge posed by antibiotic tolerance in *S. aureus* isolates associated with PJI. Using the TDtest, we observed an increase in tolerance to levofloxacin and rifampin in biofilm-embedded bacteria, to rifampin in particular. Notably, we observed a higher number of rifampin-tolerant, biofilm-embedded bacteria in strains responsible for relapsing PJIs. These findings underscore the complexity of biofilm-associated infections and raise the question of the possibility of incorporating tolerance testing into routine clinical practice. Further research is required to elucidate the molecular mechanisms driving tolerance and to explore new therapeutic strategies to overcome biofilm-related antibiotic tolerance in PJIs.

## 4. Material and Methods

### 4.1. Bacterial Isolates

The *S. aureus* isolates were obtained from a prospective multicenter study [25]. Twelve strains responsible for acute PJI cases that were managed with DAIR and the rifampin plus levofloxacin combination were selected. In order to avoid a selection bias at the time of studying the correlation of outcome and tolerance, the selection was performed randomly. Eight of these isolates were associated with a good prognosis [median follow-up 36.5 months (IQR 13.8–17.7) after debridement] and four with treatment failure due to the same *S. aureus* isolate that caused the original infection. All isolates were stored in cryovials at −80 °C (CryoBank^TM^, Copan Diagnostics Inc., Murrieta, CA, USA). Prior to conducting the experiments, each isolate was subcultured onto tryptic soy agar (TSA, BioMérieux, Madrid, Spain) plates and incubated at 37 °C for 24 h.

### 4.2. Antimicrobials and Susceptibility Testing

Levofloxacin and rifampin drug powders (Sigma-Aldrich, Madrid, Spain) were reconstituted according to CLSI guidelines [26]. Susceptibility was measured by the E-test method following EUCAST guidelines [27]. To detect bacterial persistence/tolerance to antibiotics (TDtest experiments), Mueller-Hinton agar (bioMérieux, Madrid, Spain) was used as a standard medium for testing antibiotic susceptibility.

### 4.3. Tolerance Detection Test (TDtest)

To detect tolerance, the modified TDtest, as described by Gefen et al. [16], was performed on each staphylococcal isolate studied. Briefly, a 0.5 McFarland standard was prepared for the bacterial colonies, and 100 μL of the bacterial suspension was streaked onto Mueller-Hinton agar plates. Cellulose disks that were 6 mm in diameter (Thermo Scientific Oxoid, Basingstoke, UK), preloaded with levofloxacin (1.5 μg) or rifampin (2 μg), were then added. These antibiotic concentrations were selected based on preliminary experiments with customized disks, following the approach outlined by Orit Gefen et al.: several concentrations of levofloxacin and rifampin between the amount normally found in commercial discs (i.e., 5 µg for both levofloxacin and rifampin) and 1/50 of MIC were used in a referral strain (ATCC 25923). This ensured that the concentration within the inhibition zone remained below the MIC after 24 h of incubation at 37 °C. After overnight cultivation, all antibiotic-containing disks within the inhibition zones were replaced with new, sterile cellulose disks soaked in 20 μL of 40% sterile D-glucose [16]. Growing colonies of resuscitated viable bacterial cells were observed after 48 h incubation at 37 °C. Tolerance was expressed as the number of colonies present within the inhibition zone (Supplementary Figure S1). The number of colonies in this inhibition zone were independently counted by two researchers (MA.M-C. and J.L-T.), who were blinded regarding the clinical outcome of the strain. According to previous studies using the TDtest, strains with fewer than 10 colonies would be categorized as having low tolerance, strains with 10 to 100 colonies would be categorized as having medium tolerance, and strains with more than 100 colonies within the inhibition zone would be considered as having high tolerance [16].

**Figure 5:**
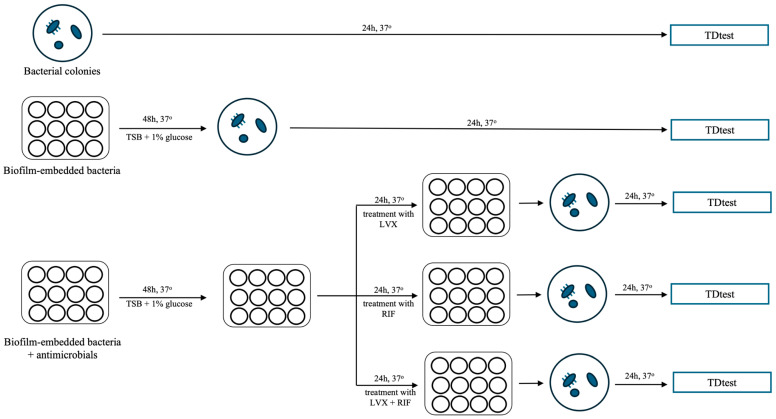
Scheme of tolerance detection test (TDtest). The TDtest was performed against (i) *S. aureus* exponentially growing (i.e., planktonic) bacterial colonies; (ii) *S. aureus* biofilm-embedded bacteria; and (iii) *S. aureus* biofilm-embedded bacteria treated with levofloxacin, rifampin, and their combination. Each specific experiment (strain and stress condition) was performed in triplicate. LVX: levofloxacin; RIF: rifampin.

The TDtest was repeated for each bacterial strain under various stress conditions: (i) with exponentially growing (i.e., planktonic) bacteria; (ii) bacteria recovered from 48 h biofilms; (iii) and from 48 h-biofilms treated with levofloxacin and rifampin for 24 h, both individually and in combination (Figure 5). Each experiment was performed in triplicate.

### 4.4. Static In Vitro Biofilm Model

Biofilms were obtained by incubating a suspension of *S. aureus* (≈10^6^ CFU/mL) for 48 h in a 12-well plate containing titanium-alloy (Ti6Al4V) disc coupons (growth area 1.57 cm^2^) in Tryptic Soy Broth (TSB, BioMérieux, Madrid, Spain) supplemented with 1% glucose (Sigma-Aldrich, Madrid, Spain), at 37 °C and 50 rpm, as previously described [28]. The medium was refreshed every 24 h. After 48 h, three coupons of each isolate were removed for the TD test. The remaining coupons were exposed to antibiotics (levofloxacin and rifampin, alone and in combination, at concentrations of 3 mg/L and 2.5 mg/L, respectively) for 24 h. We included negative biofilm controls without antibiotic, and fresh rifampin solutions were prepared before each experiment to rule out contamination and ensure antibiotic stability, following current recommendations for biofilms studies [29]. These antimicrobial concentrations are expected in bone tissue in a clinical setting [14,30]. To recover biofilm-embedded bacteria from both types of coupon (treated and untreated), the coupons were rinsed with normal saline (NaCl 0.9%, Sigma-Aldrich, Madrid, Spain) and then processed with three alternating one-minute cycles of vortexing and sonication (100 W- 40 KHz; LT-100 PRO; Tierratech, Cantabria, Spain) followed by one final vortexing step. The bacteria were then serially diluted and plated for overnight incubation at 37 °C. Bacterial density was assessed by counting the number of colony-forming units (CFU). One colony from these cultures was used for the TDtest. Results of biofilm bacterial density are normalized by the area of growth (1.57 cm^2^) and expressed as the log_10_ (log_10_ CFU/cm^2^) (the log transformation was applied to each single experiment).

### 4.5. Whole-Genome Sequencing and Gene Analysis

The whole-genome sequences of the 12 *S. aureus* isolates included in this study were retrieved from a previously published study [13]. As these isolates had already been sequenced, we used their genome data to investigate the presence of genes previously associated with tolerance and persistence, as described in the literature [12]. Sequence files were deposited at GenBank under BioProject PRJNA774351 and accession numbers: JAJHMK000000000, JAJHNB000000000, JAJHMI000000000, JAJHNN000000000, JAJHMB000000000, JAJHLP000000000, JAJHLR000000000, JAJHLS000000000, JAJHLQ000000000, JAJHMV000000000, JAJHNG000000000, JAJHNF000000000.

### 4.6. Statistical Analyses

All statistical analyses were performed using GraphPad Prism 8.2.1 software (San Diego, CA, USA). Data normality was assessed using the Shapiro–Wilk test. The Kruskal–Wallis one-way analysis of variance test was used to compare differences between multiple groups. For pairwise comparisons, the unpaired Student’s *t*-test or the Mann–Whitney U-test were used, depending on the data distribution. Associations between the presence/absence of tolerance-related genes and tolerance levels as measured by the TDtest were evaluated using *t*-tests. Spearman’s rho coefficient measured correlations between the reduction in log_10_ CFU following antibiotic treatment and the number of tolerant bacterial colonies detected by the TDtest. *p* values of <0.05 were considered significant.

## Figures and Tables

**Table 1:** Results of TDtest for levofloxacin among strains of *Staphylococcus aureus* causing prosthetic joint infection included in this study.

Strain Code ^a^	LVX MIC (mg/L)	Levofloxacin Tolerance (Absolute Number of Tolerant Colonies)			
		Non-Biofilm Bacteria	Biofilm Bacteria	Biofilm-LVX Treatment	Biofilm-LVX + RIF Treatment
Fail104	0.25	23 ± 4.4	26 ± 6.1	26.7 ± 6.8	10.3 ± 4.2
Fail401	0.125	0.7 ± 0.6	2.3 ± 2.1	3.3 ± 1.2	4.7 ± 2.3
Fail402	0.125	18.3 ± 3.1	16.3 ± 1.2	46 ± 5.6	70.7 ± 6.0
Fail409	0.25	0.7 ± 1.2	0.7 ± 1.2	66 ± 9.2	14 ± 5.3
Cure111	0.25	0.7 ± 0.6	0.7 ± 0.6	6.3 ± 0.6	9.7 ± 3.2
Cure112	0.19	19.3 ± 2.5	20 ± 2.6	18.7 ± 4.2	5.7 ± 2.1
Cure116	0.19	39.3 ± 3.8	46 ± 4.4	13 ± 4.4	10 ± 4.0
Cure208	0.19	10.3 ± 2.1	30.7 ± 4.9	3.7 ± 1.2	100
Cure804	0.125	0	0.3 ± 0.6	32.7 ± 4.9	43 ± 22.5
Cure806	0.125	4.7 ± 2.5	16.7 ± 4.7	35.7 ± 9.9	7.3 ± 1.2
Cure807	0.125	10.7 ± 2.1	16.7 ± 1.5	20.3 ± 1.5	10.3 ± 2.9
Cure808	0.19	8.3 ± 7.4	39.3 ± 1.2	39.7 ± 1.5	25.7 ± 9.7
					
**Mean ± SD ^b^**	.	11.3 ± 11.9	18.0 ± 15.5	26.01 ± 19.0	26.0 ± 30.4
**CVg ^c^**	.	104.80%	86.00%	73.20%	117.00%

MIC: minimal inhibitory concentration; LVX: levofloxacin; RIF: rifampin. Each experiment was performed by triplicated. Results of tolerance are expressed as mean ± standard deviation. ^a^ All isolates were susceptible to methicillin (MSSA); the prefix ‘Fail’ before each strain number means that this case presented with failure, considered in patients needing salvage therapy (surgical and/or medical) due to the same *S. aureus* causing the original infection; the prefix ‘Cure’ stands for strains where no failure was observed. ^b^ Mean and standard deviation (SD) of aggregated data. ^c^ Inter-strain coefficient of variation (CVg).

**Table 2:** Results of TDtest for rifampin among strains of *Staphylococcus aureus* causing prostjetic joint infection included in this study.

Strain Code ^a^	RIF MIC (mg/L)	Rifampin Tolerance (Absolute Number of Tolerant Colonies)			
		Non-Biofilm Bacteria	Biofilm Bacteria	Biofilm-RIF Treatment ^b^	Biofilm-LVX + RIF Treatment
Fail104	0.012	7.3 ± 4.9	80 ± 5.0	-	145.3 ± 22.0
Fail401	0.008	29 ± 15.6	113.3 ± 11.9	-	10.7 ± 3.8
Fail402	0.008	5.7 ± 4.2	54.7 ± 16.9	-	22 ± 9.5
Fail409	0.016	6.7 ± 5.0	144.3 ± 25.7	-	96.7 ± 6.5
Cure111	0.008	3.7 ± 1.5	25.3 ± 5.0	-	1 ± 1.0
Cure112	0.012	5 ± 4.3	19 ± 2.6	-	1.7 ± 2.9
Cure116	0.012	3.3 ± 2.1	3.7 ± 2.5	-	86.7 ± 5.0
Cure208	0.006	1.7 ± 2.1	30.7 ± 4.9	-	100
Cure804	0.008	1.7 ± 2.1	0	-	0.7 ± 1.2
Cure806	0.012	10.7 ± 1.5	54.7 ± 17.8	-	34.7 ± 1.5
Cure807	0.016	6.3 ± 0.6	17.3 ± 5.1	-	53.3 ± 5.1
Cure808	0.012	14 ± 8.9	2.3 ± 0.6	-	26 ± 9.5
					
**Mean ± SD ^c^**	.	7.93 ± 7.53	45.4 ± 46.25	.	48.2 ± 48.0
**CVg ^d^**	.	95.00%	101.80%	.	99.60%

MIC: minimal inhibitory concentration; LVX: levofloxacin; RIF: rifampin. Each experiment was performed by triplicated. Results of tolerance are expressed as mean ± standard deviation. ^a^ All isolates were susceptible to methicillin (MSSA); the prefix ‘Fail’ before each strain number means that this case presented with failure, considered in patients needing salvage therapy (surgical and/or medical) due to the same *S. aureus* causing the original infection; the prefix ‘Cure’ stands for strains where no failure was observed. ^b^ Biofilm bacteria treated with rifampin in monotherapy did not generate tolerant colonies, as all isolates developed resistance (inhibition zone 0 mm) after 24 h of antibiotic exposure in the TDtest. ^c^ Mean and standard deviation (SD) of aggregated data. ^d^ Inter-strain coefficient of variation (CVg).

## Data Availability

Genetic sequence files of bacteria have been deposited at GenBank under BioProject PRJNA774351 (see Section 4.5). Specific data on the results of the TDtest may be obtained upon request to the corresponding author.

## Supplementary Materials

The following supporting information can be downloaded at: https://www.mdpi.com/article/10.3390/antibiotics15010010/s1, Figure S1. Representative image of the TDtest showing antibiotic tolerance. Figure S2. Evolution of tolerance of the 12 *S. aureus* isolates included in the study in each stress condition.
