# Supplementary material for: A Study of Antibiotic Tolerance to Levofloxacin and Rifampin in Staphylococcus aureus Isolates Causing Prosthetic Joint Infections: Clinical Relevance and Treatment Challenges
Source: Antibiotics (Basel). 2025 Dec 20;15(1):10. doi: 10.3390/antibiotics15010010 (PMC12837759; doi:10.3390/antibiotics15010010)

Figure S1. Representative image of the TDtest showing antibiotic tolerance. Colonies inside the inhibition zone after the second step of the TDtest indicate the presence of tolerant subpopulations able to survive transient antibiotic exposure. The antibiotic disk creates a clear inhibition halo, but regrowth of tolerant colonies within this zone highlights the phenotypic tolerance of the tested *S. aureus* isolate.

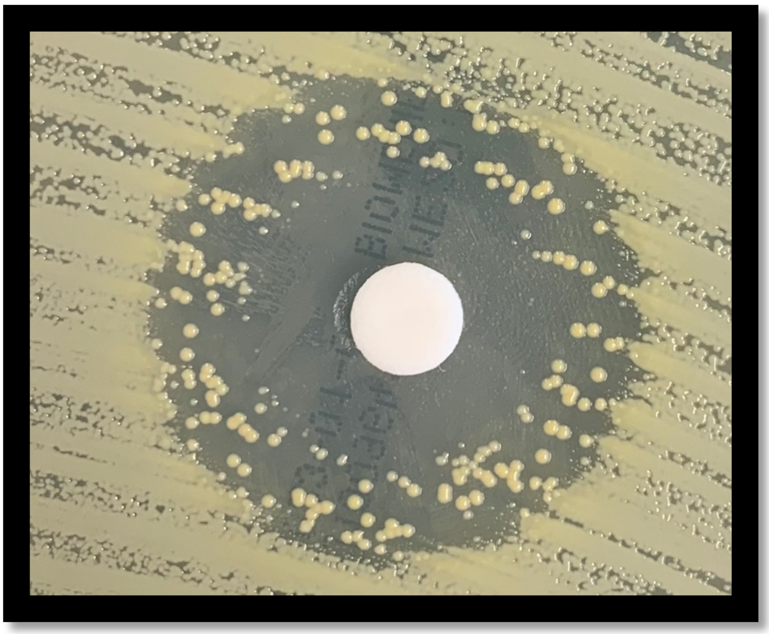

Figure S2. Evolution of tolerance of the 12 *S. aureus* isolates included in the study in each stress condition. Tolerance was expressed as the number of colonies present within the zone inhibition. Measurement of rifampin-tolerance was not possible after treatment with rifampin-monotherapy, since all strains developed resistance within 24h. Each dot represents the median of three replicates of each isolate.

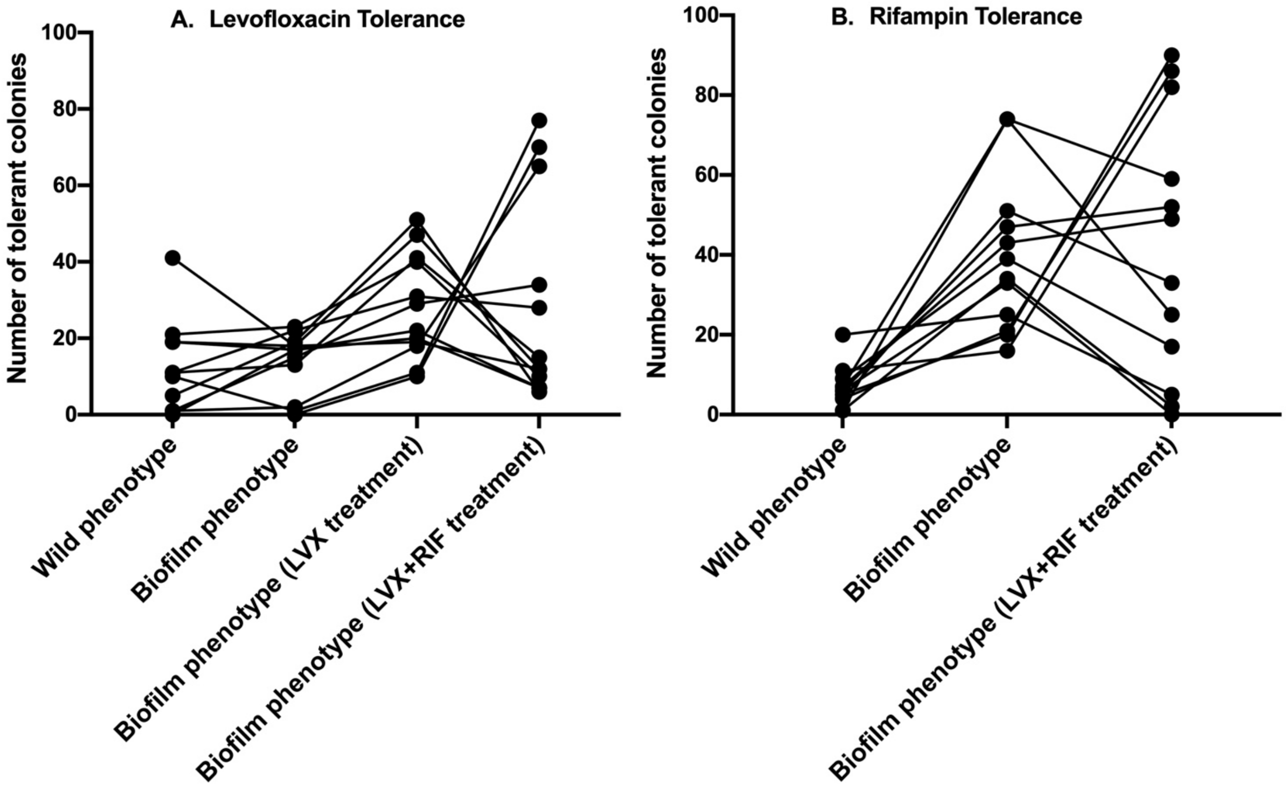

Supplement: Supplementary file 1 [file antibiotics-15-00010-s001.zip › antibiotics-3987815-supplementary.pdf]
